# Diagnostics and Treatment of Volkmann Ischemic Contracture in a Seven-Year-Old Child

**DOI:** 10.1055/s-0042-1749210

**Published:** 2022-07-19

**Authors:** Annekatrin Schulze, Jurek Schultz, Adrian Dragu, Guido Fitze

**Affiliations:** 1 Department of Pediatric Surgery, University Hospital Carl Gustav Carus, Dresden, Germany; 2 Department of Plastic Surgery, OUPC, University Hospital Carl Gustav Carus, Dresden, Germany

**Keywords:** Volkmann ischemic contracture, compartment syndrome, tenotomy, supracondylar fracture, children

## Abstract

A 7-year-old boy presented 6 weeks after open reduction and crossed Kirschner wire (K-wire) fixation of a supracondylar humerus fracture. Previous treatments had restored skeletal anatomy without documented complications. However, the patient would not move the entire arm, including his forearm and hand. Any passive movement led to anxious adverse reactions, and there was partial numbness of all fingers. After intensive physio- and occupational therapy supported by nerve stimulation and psychological counseling, anxiety-related functional deficits of the shoulder and elbow resolved to reveal the severe Volkmann contracture of the right hand developed fully. Electroneurography, X-ray, magnetic resonance imaging of the forearm, and ultrasonography showed nonfunctional ulnar and a partially disturbed radial motor nerve distal to the elbow along with damaged flexor muscles of the forearm after compartment syndrome. In addition, damage to the median nerve at the elbow level was diagnosed. After intense conservative therapy, we partially resected fibrotic fascia of the superficial flexor compartment, freed ulnar and median nerves, and performed staircase-like releases of tendons and tenotomies. We achieved a full range of motion of all fingers and markedly improved the range of motion of the wrist. The Disabilities of the Arm, Shoulder and Hand scores for function improved from 80 to 16 at the 2-year follow-up postoperatively, but some impairments of fine motor function persisted. Subtle symptoms of a developing compartment syndrome need to be recognized. Overlooked and untreated, a consecutive Volkmann contracture can turn the extremity nonfunctional. Intensive physical, psychological, and surgical therapy in a specialized center can restore function but requires endurance and perseverance throughout the lengthy recovery.

## Introduction

Supracondylar fractures of the humerus (SFH) represent 6.5% of pediatric fractures and 55 to 80% of elbow fractures in children, predominantly in 5- to 7-year-old children. 1 2 Especially in severely displaced fractures (Gartland type III), direct neuronal and vascular injuries may occur. 3 4 Additionally, increased pressure from swelling and hematoma after soft-tissue trauma can lead to an acute compartment syndrome (ACS) that occurs in 0.1 to 0.3% of children with SFH, while the volar superficial and deep flexor compartments are most commonly affected. 5 6 7 8 Since Baumann's vertical suspension and plastering have been replaced by percutaneous Kirschner wire (K-wire) osteosynthesis as the primary choice in the management of SFH, the incidence of the ACS has declined drastically and is, therefore, rarely encountered anymore by the medical staff. 6 7 9 Typical clinical signs of ACS of the forearm in adults are summarized as the six “P's”: pain, positive passive stretch test, paresthesia, paralysis, painful tense forearm, and at times, a pulseless limb. 10 In children, however, the primary symptoms are rather summarized as the three “A's”: anxiety, agitation, and increased analgesic requirements due to pain out of proportion along with pain on passive stretching. 11 Increasing pain and firmness of a muscle compartment on palpation over time may indicate the presence of a sub-ACS. But even in the absence of pain, a compartment that is rigid on compression with impairment of peripheral blood perfusion can lead to the diagnosis of silent ACS. 12 In any case, ACS needs to be recognized and treated immediately by fasciotomy to prevent the development of Volkmann ischemic contracture (VIC). VIC was first described by Richard von Volkmann in 1881 as a loss of function due to tissue damage following ACS. 13 The symptoms of VIC of the volar compartment of the forearm are shown in Table 1 . 9 Physiotherapy, including passive stretching of contracted muscle fibers and joints and splinting to oppose further contractions, has to start immediately. 8 Surgical treatment choices include skin release and Z-plasty for mild contractures, the release of secondary nerve compression, muscle slides, and tendon lengthening in moderate contractures, and finally, tendon transfers or free-tissue transfers as well as salvage procedures for severely contracted or neglected extremities. 14 As a consequence of this disorder's heterogeneous clinical presentation, every patient requires a customized therapy strategy to achieve alleviation from handicap to the greatest possible extent. We present the following case to outline our treatment approach and its outcome in a patient with a severe VIC.

**Table 1:** Tsuge classification—Volkmann ischemic contracture

Type	Affected muscles	Neurological finger position
Mild	M. flexor digitorum profundus	None or minimal loss of sensibility, contracture of two or three fingers
Moderate	M. flexor digitorum profundus, m. flexor pollicis longus and parts of superficial flexor muscles	Loss of sensibility in (parts of) hand all fingers, thumbs and wrists affected
Severe	All flexor muscles and parts of extensor muscles	Serious loss of sensibility and motor function, claw hand

## Patient Information

### Clinical Findings

A 7-year-old boy fell on his right arm while playing and, subsequently, another child stepped on the same arm. A local pediatric trauma surgeon in an adult trauma unit correctly diagnosed the Gartland III SFH and performed an open reduction via a dorsal approach followed by crossed K-wire osteosynthesis. The ulnar nerve was visualized and found unharmed during surgery. A dorsal noncircumferential cast was administered in 90 degree flexion of the elbow at the end of the surgery. Postoperatively, a sensory and motoric deficit in the area covered by the ulnar nerve arose without any signs of further impairment of perfusion, motion, or sensibility. The radial pulse was palpable at all times. The boy complained about severe pain over a prolonged time. In the following weeks, the range of motion of the right hand deteriorated drastically. Nerve conduction velocity was measured 4 weeks after the injury. All three nerves in the forearm were affected with a complete absence of conduction in the ulnar and median nerves. The K-wires and the cast were removed before in-patient rehab 6 weeks after the injury. After failing to improve within the first week of rehab, the child was submitted to our child trauma reference center, where the patient presented with a relieving posture of the entire right arm.

The radial and ulnar pulses were palpable, and the capillary refill showed no delay. Upon assessment of the motoric functions, the shoulder muscles M. deltoideus and M. trapezius were already hypotrophic. The elbow was held in a 20 degree flexed position with a complete lack of active motion. Passively, 90 degree flexion was possible under intensive distraction. In the wrist, the patient could only carry out wiggling motion of a maximal deflection of 20 degree, and the hand was mainly held fixed in a 20 degree flexed position. A minimal active extension of the wrist excluded a complete deficit of radial motor nerve. The fingers could not be moved and displayed flexion in all joints, and passive extension of the fingers required force and again intensive distraction of the child. The sensibility was only absent distally to the metacarpophalangeal joints. Additionally, the thenar presented with dysesthesia. In summary, no isolated or combined nerve lesion explained our clinical findings.

### Medical, Family, and Psychosocial Background

The patient, a boy of lean stature with a gluten-free diet following the diagnosis of celiac disease, has had no prior surgery other than circumcision. The otherwise healthy and well-integrated student in grammar school had many interests including sports (cycling, swimming, and karate) and playing the violin. There was no family history of any medical condition. The patient lived in a household with his parents and a 10-year-old healthy sister.

## Diagnostic Assessment

Our primary clinical findings were inconclusive and did not point to any specific nerve dysfunction. Besides, the patient showed a severe adverse reaction to the right upper limb's touch and motion, indicative of an additional psychological cause. According to the Budapest criteria for the diagnosis of a complex regional pain syndrome, the diagnosis is made when there is continuing pain disproportionate to the inciting event and certain alterations in three out of the four categories sensory, vasomotor, sudomotor, and motor/trophic. 15 Our patient presented with hyperesthesia, temperature asymmetry, and beginning hypotrophy of the affected limb alongside the severe pain so that the criteria were fulfilled.

**Fig. 1:**
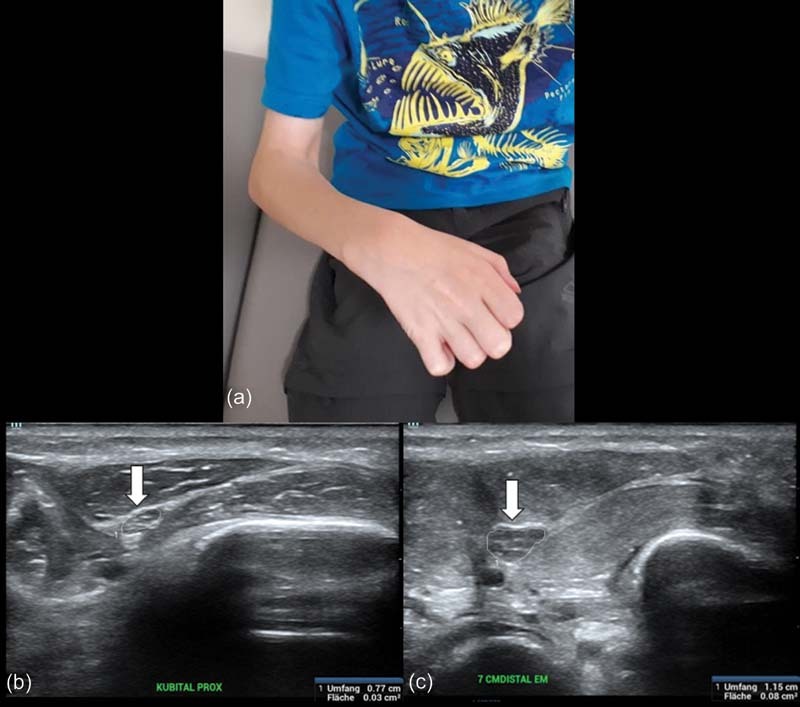
( **a** ) Clinical picture of Volkmann ischemic contracture preoperatively and ( **b and c** ) nerve ultrasonography of the ulnar nerve at cubital and forearm level.

Diagnostics included a magnetic resonance (MR) angiography, an X-ray of the forearm, repeated nerve conduction velocity assessments, and sonography of the nerves ( Fig. 1b, c ) and muscles of the forearm. They revealed an injury to the median nerve at the elbow joint and muscle deterioration in both flexor muscle compartments with the ulnar nerve's affection 7 cm distally to the ulnar epicondyle following the suspected compartment syndrome. The brachial, radial, and ulnar arteries did not show any sign of insufficiency. After an intensive course of conservative therapy, the shoulder and upper arm's muscular deficit resolved and the range of motion of the elbow joint improved significantly. Subsequently, the typical Volkmann contracture developed and could be observed 18 weeks postinjury ( Fig. 1a ).

## Therapeutic Interventions

After the cast and metal removal 6 weeks postinjury, the patient attended an intensive inpatient rehabilitation program for 5 weeks. It included physiotherapy and occupational therapy under analgesics to extend the active and passive range of motion of the affected limb supported by psychological consultations. To prevent worsening of the contracture of the wrist and hand, a volar splint was fitted. Additionally, physical therapy was administered for sensory stimulation and tonus regulation. At the end of rehab, the patient had fully restored function and strength of the affected shoulder and upper arm, extended range of motion of the elbow to an extension/flexion of 0–40–100, and improved pronation to 60 degree. However, the clinical picture of VIC with severely limited extension of the wrist and contracture of the fingers in flexion developed fully. Intensive conservative therapy was continued in an outpatient setting adding further modalities such as electrostimulation, osteopathy, therapeutical swimming, and BEMER physical vessel therapy. 16 In the meantime, diagnostics were completed.

**Fig. 2:**
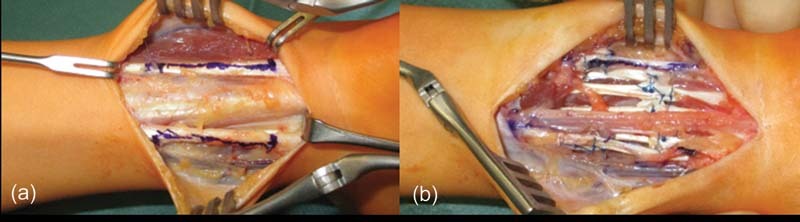
Steps of the surgical procedure after neurolysis and stepwise tenolysis and tenotomies.

When conservative therapy failed to improve the clinical picture further, the surgical procedure was planned and performed by an interdisciplinary team of pediatric and hand surgeons. For optimal vision, we operated with a tourniquet and magnifying glasses. We excised severely fibrotic and thickened fascia tangentially via a proximal incision the size of the distal incision ( Fig. 2 ) in an attempt to improve muscle function, as it has proven to be successful on the lower extremity. In this case, it, unfortunately, did not improve motility. We then created access to the distal volar forearm. Here, the median and ulnar nerves ( Fig. 2a ) alongside the radial and ulnar arteries were microsurgically depicted and spared. We tenotomized M. palmaris longus and Mm. flexors digitorum superficialis D2 to 5 in addition to the stepwise elongation of the tendons of Mm flexor carpi ulnaris and flexor carpi radialis as well as Mm. flexor digitorum profundii D2 to 5 ( Fig. 2b ). Finally, an elongation of the M. flexor pollicis longus tendon allowed for full extension of all fingers and wrist. The partially necrotic and fibrotic flexor muscles were spared during the surgery to prevent further damage. After surgery, a Kleinert splint was custom-fitted to enable early postoperative training and maintain tendon mobility. Meanwhile, the patient proceeded with occupational therapy.

## Follow-up and Outcomes

Immediately after the intervention, the patient demonstrated an excellent range of motion of the fingers on active extension and passive flexion by the Kleinert splint. 17 Furthermore, the patient achieved a completely unimpaired range of motion of the elbow and forearm and subsequently improved the right upper limp's flexibility and strength.

In the final clinical assessment and for the time being, the patient felt content with the outcome. Minor residues were no longer noticeable, and the slightly diminished strength in finger flexion did not bother. The range of motion of the hand and wrist was close to normal. The Disabilities of the Arm, Shoulder, and Hand scores throughout the recovery process improved from 80 to 16 in overall function and from 100 to 0 in sports. With the help of his very supportive and encouraging family, who actively took part in the therapy, the patient has now started a career in swimming, where he already competes on regional and national levels.

## Summary and Discussion

In this case of a 7-year-old patient with a severe SFH treated by open reduction and K-wire fixation plus dorsal plaster cast, the development of a sub-ACS was missed and therefore left untreated. The subsequent psychological affections further complicated clinical examination and diagnosis upon referral to our pediatric trauma center. Nonetheless, the appropriate initial therapy was commenced in a rehabilitation clinic that comprised physiotherapy with passive stretching of the affected flexor tendons and muscles under analgetic therapy and active training of the affected and unaffected muscles of the arm and shoulder as well as occupational therapy and psychological counseling. In the meantime, further diagnostic measures were taken to elucidate the cause of the symptom complex including MR angiography, repeated electroneurography, and specialized ultrasonography of the limbs' nerves. They revealed damage to the median nerve at the level of the cubital fossa as a consequence of the initial injury and affection of the flexor muscles and ulnar nerve as well as partial radial nerve at the level of the forearm due to ACS prompting the clinical picture of VIC.

A multidisciplinary case conference involving neurosurgeons, hand surgeons, and orthopedic surgeons discussed the diagnostic and further therapeutic steps. These included a surgical intervention after the conservative efforts were exhausted. Surgical options vary according to the severity of the condition. They include skin release and Z-plasty for mild to moderate contractures and the release of secondary nerve compression, muscle slides, tendon lengthening, tendon or free-tissue (e.g., gracilis muscle) transfers, and salvage procedures for severely contracted or neglected extremities. 14 Unfortunately, an earlier surgical intervention after referral to us and before the completion of the deterioration process would not have been promising to yield a more fortunate result. 18 In our case a combination of fascia release, neurolysis, tenotomies, and tendon elongations was performed and postoperatively only minor deficits persisted. Sadly, the extension of the wrist, which should be the first goal of therapy, 14 could not be maintained completely throughout time, which is most likely due to the M. palmaris longus tendon's persistent adherence, despite the tenotomy performed. A persisting and impairing fine motor function deficit remained mainly due to the small finger muscles' affection (Mm. lumbricales) by the initial lack of perfusion during the ACS, which cannot be improved further by surgical therapy.

We conclude that VIC resulting from an overlooked ACS after SFH in children is a potentially devastating condition requiring a long course of intensive conservative and surgical treatment. However, despite all efforts *restitutio ad integrum* may never be achievable as is the case with our patient. In the pediatric population, clinical examination may sometimes be challenging due to adverse behavior and pain. Furthermore, circular plaster casts are regularly administered in the treatment of fractures and present an additional risk for the development of a sub-ACS. They may also limit the access to the limb for clinical assessment and, therefore, delay diagnosis. Health care practitioners must recognize the three A's anxiety, agitation, and increased analgesic requirement as the symptoms of ACS and upon suspicion, compartment pressure measurement should be done immediately followed by fasciotomy to prevent further damage to muscular and nerve structures if necessary. 10 19 Even in the absence of pain and the presence of symptoms such as severe swelling and impaired or inconspicuous neurovascular function, a possible silent ACS needs to be taken into account and treated timely. 11

Despite the remaining limitations mainly consisting of the lack of strength in finger flexion and an impairment of full extension in the wrist, our patient leads a happy life pursuing his hobby swimming, where he feels no restrictions at all and competes on a national level. We are content with the success of our interdisciplinary team effort and wish our patient the best for his future.

## Notes

Importance and Newinsight for the Pediatric Surgeon Volkmann Ischemic contracture is a rare, but debilitating condition following supracondylar fractures in children requiring intensive interdisciplinary conservative and surgical treatment to relieve symptoms. Early signs of the preceding compartment syndrome consisting of unusually intense, prolonged pain and secondary nerval deficits early after trauma should to be recognized to prevent its development.
